# Looking beyond community structure leads to the discovery of dynamical communities in weighted networks

**DOI:** 10.1038/s41598-022-08214-z

**Published:** 2022-03-16

**Authors:** Chad Nathe, Lucia Valentina Gambuzza, Mattia Frasca, Francesco Sorrentino

**Affiliations:** 1 Department of Mechanical Engineering, University of New Mexico, Albuquerque, NM 87131 USA; 2 Department of Electrical, Electronics and Computer Science Engineering, University of Catania, Catania, Italy

**Keywords:** Mathematics and computing, Applied mathematics

## Abstract

A fundamental question is whether groups of nodes of a complex network can possibly display long-term cluster-synchronized behavior. While this question has been addressed for the restricted classes of unweighted and labeled graphs, it remains an open problem for the more general class of weighted networks. The emergence of coordinated motion of nodes in natural and technological networks is directly related to the network structure through the concept of an equitable partition, which determines which nodes can show long-term synchronized behavior and which nodes cannot. We provide a method to detect the presence of nearly equitable partitions in weighted networks, based on minimal information about the network structure. With this approach we are able to discover the presence of dynamical communities in both synthetic and real technological, biological, and social networks, to a statistically significant level. We show that our approach based on dynamical communities is better at predicting the emergence of synchronized behavior than existing methods to detect community structure.

## Introduction

The study of symmetries has led to an understanding of many important problems in physics, including the formulation of the standard model and general relativity^1–4^, chemistry^5^, and biology^6^, as symmetries are widespread in the natural world. Symmetries have also been found to affect the structure of many biological, technological, and social systems described as networks^2,7–15^. However, for the most part, the existing literature has only focused on unweighted networks. Given that most real networks are weighted and that the edge weights provide key information to understanding the network structure and dynamics^16–19^, it becomes important to define and characterize ‘approximate symmetries’ and thus ‘approximate clusters’ in weighted networks. In this paper we first introduce the concept of approximate clusters and then look for the presence of these approximate clusters in real network datasets. Being able to find these approximate clusters is important because these will be the clusters of nodes that in a weighted network can produce approximate cluster synchronization^20^ or approximately equal time-averaged dynamics^21^. We thus call these clusters ‘dynamical communities’, as opposed to fixed communities corresponding to the network community structure^22^, where a community is defined as a set of nodes that are densely connected with one another but sparsely connected with other communities.

In the case of exact symmetries, the set of network nodes is partitioned into disjoint sets of clusters, with all nodes that are symmetric to one another forming a cluster. It has been found that these clusters are linked with the ability of networks to cluster-synchronize^12,13^ and to achieve similar time-averaged dynamics^21^. A generalization is that of equitable clusters that characterize synchronization for nodes that are not necessarily related by symmetries but receive the same total amounts of inputs from their neighboring nodes in different clusters^14,23,24^. Similar to symmetric nodes, nodes in the same equitable clusters are also associated by an equivalence relation and coordinated motion of clusters of nodes is only possible when the clusters form an equitable partition^2,7,8,10,15,23^. It has also been shown that approximate cluster-synchronization can be observed when the network structure possesses approximate symmetries^20^. While there are a number of tools to detect approximate symmetries from different disciplines^11,25–27^, the problem of identifying clusters of nodes in weighted networks where each cluster is formed of almost equivalent nodes has not received much attention. An inherent difficulty is due to the fact that the definition of an approximate symmetry will lead to loss of the transitive property, i.e, two pairs of nodes, *i* and *j* and *j* and *h*, may be approximately symmetric, without nodes *i* and *h* being approximately symmetric. In other words, the set of approximate symmetries with the composition operation is not closed, thus they do not form a group. This fundamental challenge motivated us to follow an alternative approach, where instead of looking for approximate symmetries, we directly look for approximate ‘nearly equitable clusters’.

Clustering approaches have been widely applied to the field of network science. The most notable example is that of the network community structure^22^, where a community is defined as a set of nodes that are densely connected with one another but sparsely connected with other communities. Though this provides an important characterization of a complex network, the presence of community structure does not imply in any way that the elements of these communities display similar time-evolutions when the network equations are evolved in time. Our goal in this paper is specifically to look for clusters of nodes that are both structurally and dynamically nearly equivalent, which is required condition for the emergence of approximate synchronization^20^ or approximately equal time-averaged dynamics^21^. We call such clusters dynamical communities.

## The case of exact equitable clusters

In the presence of a network topology in which the weights come from a finite set (labeled graphs), a *minimum balanced coloring* can be calculated using the Belykh and Hasler (BH) algorithm^28^. We start from knowledge of the adjacency matrix *A*, which describes the topology of a network, i.e., $A_{ij}>0$ if node *j* is connected to node *i* and $A_{ij}=0$ otherwise. We emphasize that the network is generically directed, and so the adjacency matrix asymmetric. Given *A*, this algorithm computes a cluster partition of nodes that uses the minimum number of clusters needed. We call these the *true clusters* and label $k^*$ the number of such clusters. This efficient polynomial algorithm is described below.

1. At first, all the nodes, labeled here as $1,\ldots ,N$ are placed into one cluster so that $k=1$ and ${\mathcal {C}}=\{{\mathcal {C}}_1\}$ with ${\mathcal {C}}_1=\{1,\ldots ,N\}$. Then a process of refinement of the partition is started.
2. The $N \times K$-dimensional matrix *P* is created, whose entries are the cluster degrees $P_{ij}$ of node $i=1,\ldots ,N$ to each cluster $j=1,\ldots ,K$,
  1 $$\begin{aligned} P_{ij} = \sum _{\ell \in {\mathcal {C}}_j}A_{i\ell }. \end{aligned}$$
  The cluster degree is the overall coupling that node *i* receives from the nodes in cluster *j*.
3. Based on the information contained in the matrix *P*, a new cluster partition is built, where nodes having equivalent rows are placed in the same cluster. Note that exact equivalences are transitive, that is, if nodes *i* and *j* are equivalent and so are *j* and *h*, then also *i* and *h* are equivalent. The matrix *P* reflects this property in that if its row *i* is equal to its row *j* and its row *j* is equal to its row *h*, then also rows *i* and *h* are equal.
4. Steps 2) and 3) are repeated with the new cluster partition. This process iterates until the cluster partition cannot be further refined, then we set $k=k^*$ where $k^*$ represents the number of the true clusters.

## The case of nearly equitable clusters

The BH method of “Section The case of exact equitable clusters” works when symmetries within a network are exact, however it is not designed to detect approximate symmetries or equivalence relations. In order to address this issue, we propose a variation of the BH algorithm which returns *nearly equitable clusters* or *dynamical communities*. This method uses a top-down methodology similar to divisive hierarchical clustering^29^, in that we start with $k=1$ cluster, then break this cluster down specifying $k \rightarrow k+1$ with each iteration until $k=N-1$. We outline our process below. Steps 1) and 2) are the same as described for exact symmetries. They are followed by steps 3) and 4) below:

(3) Create a dissimilarity matrix, *D*, which describes the difference in cluster degrees between node *i* and node *j*. *D* is a symmetric matrix with a main diagonal of zero, and size $N\times N$. Each entry $D_{ij}=D_{ji}$ is equal to the Euclidean norm of the difference in cluster degrees from node *i* to node *j*, that is:

2 $$\begin{aligned} D_{ij} = \sum _{i=1}^N \sum _{j=1}^N \Vert P_{i} - P_{j}\Vert , \end{aligned}$$

where by $P_i$ ($P_j$) we indicate row *i* (*j*) of the matrix *P*. Note that the fact that $D_{ij}< \alpha$ and $D_{ih}<\alpha$ will not necessarily imply that $D_{jh}<\alpha$. It is also unlikely that there will be zero entries indicating exactly equivalent nodes. Hence, we apply *k-medoids* clustering^30^ to the *D* matrix where we specify the number of clusters as *k*. In the case of the first iteration, we will specify $k=2$, we then increase *k* by one $k \rightarrow k+1$. At each iteration, from the solution given by *k-medoids*, we now have a cluster partition which contains one more cluster than in the previous iteration.

(4) Repeat steps 2) and 3) for any possible number of clusters until $k=N-1$. Note that we are excluding the trivial case $k=N$ where each cluster contains a single node.

To optimize the cluster partition generated by *k-medoids*, we utilize the following process:

- Run *k-medoids* several times using the *kmeans++* starting algorithm for initial medoids locations.
- Then choose the cluster partition which yields the lowest average intra-cluster to medoid distance.

**Figure 1:**
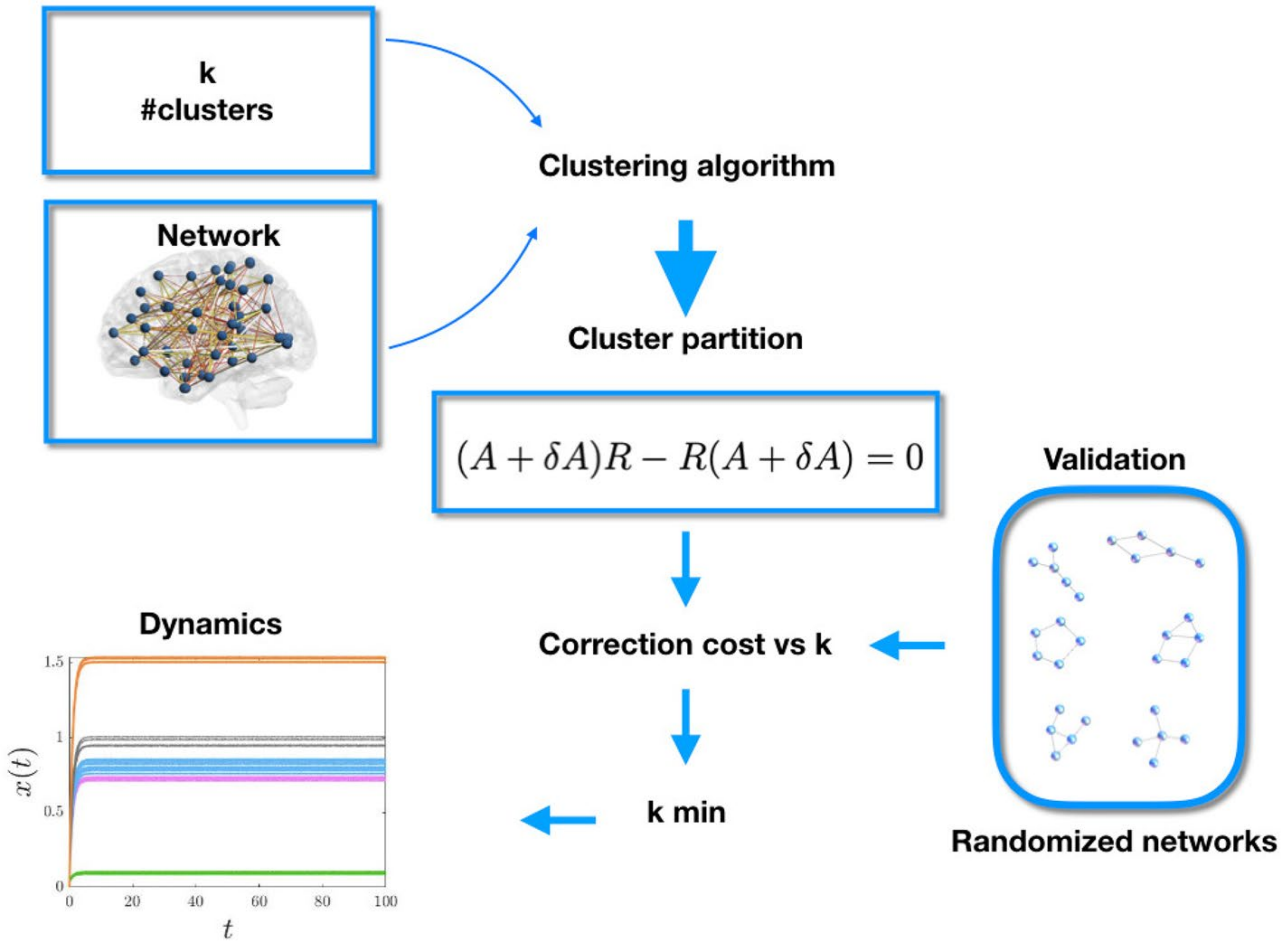
Schematic representation of the method to determine and validate an approximate cluster partition for a network. The clustering algorithm of “Section The case of nearly equitable clusters” is fed with data on a network (in particular, its adjacency matrix) and a given number of clusters, *k*. This algorithm determines an approximate cluster partition for which we calculate the corresponding projection operator $E_H$. In turn, this is used to calculate $\delta A$ from Eq. (6) with $R=E_H$. The correction cost $\psi =\Vert \delta A\Vert$ is then contrasted for statistical significance with the result of randomized graphs obtained from the original network, swapping the links.

The above clustering algorithm produces a cluster partition to which we can associate a *correction cost*, which we explain below. Our general methodology is illustrated in Fig. 1, which visually depicts the process used to create and validate a cluster partition.

In order to quantify how close to an equitable partition a given cluster partition is (and hence how much ‘approximated’ a solution of the modified BH algorithm is), we associate with each solution a correction cost, i.e., a cost of correcting the matrix *A* so that it displays the desired clusters. If we start with a permutation matrix, *R*, which describes the symmetry of the adjacency matrix, *A*, then the equation:

3 $$\begin{aligned} AR-RA=0 \end{aligned}$$

is satisfied. If the *R* matrix does not commute with *A*, then,

4 $$\begin{aligned} AR-RA\ne 0. \end{aligned}$$

Following Ref.^31^, we assume that we want to retain the matrix *R* but ‘correct’ the matrix *A* in order to make Eq. (3) hold true, and we write,

5 $$\begin{aligned} (A+\delta A)R - R(A+\delta A) = 0 \end{aligned}$$

where $\delta A$ is the perturbation matrix which must be added to *A* in order to satisfy the commuting property. Eq. (5) has solution^31^,

6 $$\begin{aligned} \delta A = (I\otimes R - R^{T}\otimes I)^{+}\text{ vec }({- R A + A R}) \end{aligned}$$

where by the notation $M^+$ we denote the Moore-Penrose inverse of the matrix *M*, and $\text{ vec }(M)$ indicates vectorization, i.e., the linear transformation which converts the matrix *M* into a column vector $\text{ vec }(M)=[M_{1,1} M_{2,1} \ldots M_{N,1} M_{1,2} \ldots M_{N,N}]^T$. We can now define the correction cost as,

7 $$\begin{aligned} \psi = \Vert \delta A\Vert \end{aligned}$$

where $\Vert M \Vert$ is the Euclidean norm of the matrix *M*. We can solve this problem for any desired cluster partition. In what follows we will compute the correction cost associated with an equitable partition by setting $R=E_H$, where $E_H=E(E^T E)^{-1}E^T$ is the projection operator and *E* is the indicator matrix associated with the given equitable partition, i.e., $E_{ij}=1$ if node *i* belongs to cluster ${\mathcal {C}}_j$, and $E_{ij}=0$ otherwise.

Using the modified BH algorithm described above, we create a cluster partition for each value $k=2,\ldots ,N-1$. The closer the cluster partition is to the underlying symmetries of *A*, the lower the correction cost will be. In this way, we characterize $\psi$ as a function of *k* in order to determine how the correction cost changes with different partitions. Later on, this will help us determine which value of *k* should be used to reconstruct the *approximate clusters*. Since the correction cost will trivially decrease as *k* is increased (due to the correction cost being higher for a larger number of equivalence relations), we introduce the scaled correction cost ${\hat{\psi }}(k)$ which is defined as

8 $$\begin{aligned} {\hat{\psi }}(k)=k\psi (k). \end{aligned}$$

This rewards partitions with larger clusters and penalizes partitions with smaller clusters. In what follows, we will typically compute both $\psi (k)$ and ${\hat{\psi }}(k)$ and use both indices to select a number of clusters *k*.

As mentioned in the introduction, cluster partitions affect the dynamical behavior of a network. This is true independent of the particular dynamics at the network nodes. To show this, we consider two examples: consensus and synchronization dynamics. Let us start with the simple case of consensus dynamics described by the equation,

9 $$\begin{aligned} \begin{aligned} {\dot{x}}(t)=&(A- \rho I) x(t) +\delta ,\\ x(0)=&0, \end{aligned} \end{aligned}$$

where the *N*-dimensional vector $x(t)=[x_1(t),x_2(t),\ldots ,x_N(t)]$ represents the state of each one of the *N* network nodes, the *N*-dimensional vector $\delta$ is a time-constant forcing term. In what follows, we assume $\delta$ to be a vector whose entries are all ones. By the assumption that the scalar $\rho$ is large enough to make the matrix *A* Hurwitz, the time evolution ${x}(t)=(A- \rho I)^{-1}[e^{(A-\rho I)t}-I] \delta$ and the steady-state solution $x^{ss}=-(A- \rho I)^{-1}\delta$. In the case of exact equitable partitions, it can be shown that nodes in the same cluster follow exactly the same time evolution of the consensus dynamics^32^. For the case of a nearly equitable node partition, by assuming stability, we expect almost synchronized consensus dynamics. This will be shown in what follows by plotting cluster-color-coded curves corresponding to the time evolution of each node $x_i(t)$, $i=1,\ldots ,N$.

To illustrate our method, we begin by considering synthetic networks. In particular, we start with a network that has a set of true clusters, which we perturb with increasing noise. Under these conditions, we expect that for low noise the structure of the cluster partition is somehow preserved, giving rise to an approximate cluster partition, up to a scenario in which the noise is so large that no partition can be retrieved. In particular, here we consider two types of perturbations applied to synthetic networks, called Type I and Type II:

10a $$\begin{aligned} A =&A_0 + \epsilon Q \circ A_0 \quad \text{(Type } \text{ I) } \end{aligned}$$

10b $$\begin{aligned} A =&A_0 + \epsilon Q \quad \text{(Type } \text{ II) }, \end{aligned}$$

where $A_0$ is the synthetic network adjacency matrix, $\epsilon$ is the magnitude of perturbation and *Q* is a full matrix composed of elements randomly drawn from a standard normal distribution. The symbol $\circ$ indicates entry-wise product (the Hadamard product), so that in Eq. (10a), a perturbation is only applied to the existing nonzero entries of $A_0$, while in Eq. (10b), a perturbation is applied to all node pairs.

**Figure 2:**
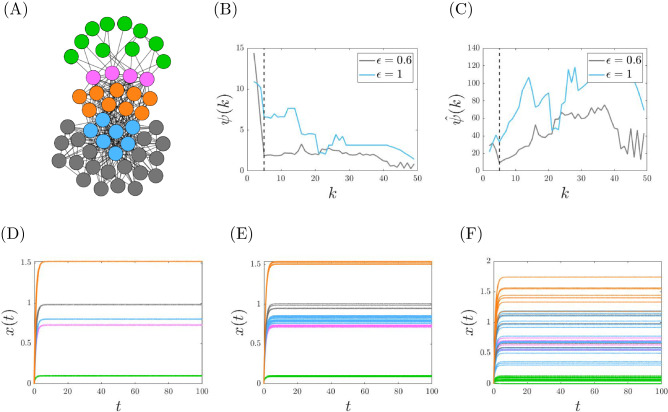
Correction cost for a synthetic network with $N=50$ nodes. (**A**) Structure of the network. In the absence of perturbations this network has $k^*=5$ true clusters (dashed lines in panels B and C). Nodes are colored differently according to the true cluster to which they belong. (**B**) Correction cost $\psi$ versus *k* for two magnitudes of perturbation. (**C**) Correction cost ${\hat{\psi }}$ versus *k* for two magnitudes of perturbation. (**D**–**F**) Consensus dynamics as described in Eq. (9) with a type I perturbation with respective magnitudes $\epsilon =0$, $\epsilon = 10^{-1}$, and $\epsilon =10^{0}$.

An example of the analysis of a synthetic network with $N=50$ nodes is illustrated in Fig. 2. In this case, the unperturbed network has $k^*=5$ true clusters (Fig. 2A). For the perturbed networks the original cluster partition with $k^*=5$ is no longer exact. However, it is an approximate cluster partition as can be seen from the correction cost $\psi$ and the scaled correction cost ${\hat{\psi }}$ in Fig. 2B,C. For a small enough perturbation, both curves display a minimum at $k=5$ that is local for $\psi$ and global for ${\hat{\psi }}$. When the perturbation is too large for the symmetries to be uncovered (such as for the value of $\epsilon =1$ shown in panels B and C), these minimums are lost. The existence of an approximate cluster partition is reflected into the dynamical time-evolution of the network nodes. To illustrate this, in Fig. 2D–F we have considered three perturbations $\epsilon$ of increasing magnitude and integrated Eq. (9) with the corresponding adjacency matrix obtained in the three scenarios. We note that, when the network is unperturbed, the cluster partition is exact and the state variables converge to consensus values mirroring the exact cluster partition (Fig. 2D, $\epsilon =0$). For $\epsilon =10^{-1}$ the cluster partition becomes approximate, with a small associated correction cost. The presence of this approximate cluster partition affects the dynamics of the network, as the state variables of the nodes in the same dynamical community are now not seen to overlap anymore, though they tend to remain close to one another (Fig. 2E, $\epsilon =10^{-1}$). The spread becomes larger for a larger magnitude of the perturbation (Fig. 2F, $\epsilon =10^0$). A similar behavior is observed in the case of synchronization dynamics, which is shown in the SI.

For the synthetic network of Fig. 2 we have also studied the case where the nodal dynamics are oscillatory and in particular chaotic. In this case the network evolves to reach a cluster synchronization state which mirrors the nearly equitable cluster partition. We illustrate here the case of a network of coupled Rössler oscillators, for which the governing equations are,

11 $$\begin{aligned} \begin{array}{l} {\dot{x}}_{i} = -y_{i} -z_{i} \\ {\dot{y}}_{i} = x_{i} + a y_{i} + \gamma \sum \limits _{j=1}^N A_{ij} y_j\\ {\dot{z}}_{i} = b + z_{i} (x_{i} -c)\\ \end{array} \end{aligned}$$

$i=1, \ldots , N$, where the parameters of the isolated nodal dynamics are equal to $a = b = 0.2$ and $c = 9$, which produces uncoupled chaotic dynamics. The value of the coupling coefficient is set to $\gamma =0.01$. The elements of the perturbed matrix *A* are obtained following the procedure discussed in Sec. 1 of the SI, in particular, the parameter $\sigma$ in Eq. (2) of the SI has been set to $\sigma =0.002$.

**Figure 3:**
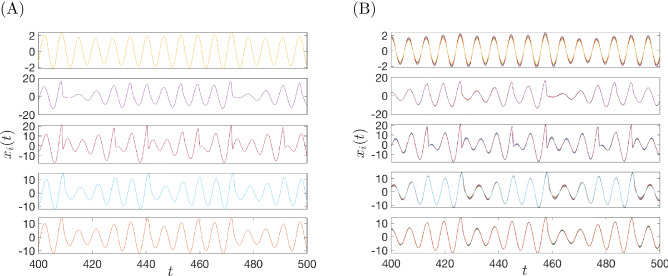
Dynamics of Rössler oscillators coupled according to the synthetic network in Fig. 2 : (**A**) when the network is unperturbed exact clusters synchronization is observed; (**B**) when the network is perturbed approximate cluster synchronization appears. From top to bottom, state variables $x_i(t)$ for $i=1, \ldots , 10$ (first panel); $i=11, \ldots , 14$ (second panel); $i=15, \ldots , 21$ (third panel); $i=22, \ldots , 41$ (fourth panel); $i=42, \ldots , 50$ (fifth panel).

Figure 3 shows the time evolution of the *x* variable of the network nodes, both for the unperturbed and perturbed cases. In the unperturbed case, the network has $k^*=5$ true clusters, correspondingly, the node variables group into $k=5$ clusters with different oscillatory dynamics (Fig. 3A) For the perturbed network the clusters are no longer exact, and likewise, neither is the nodal synchronization dynamics within the clusters. However, clusters can still be clearly identified from the nodal dynamics (Fig. 3B).

## Statistical significance analysis

Let us now consider the case of real weighted networks for which it is not known whether an underlying cluster partition exists (and what the dynamical communities are.) Next we present a method that takes as an input the weighted adjacency matrix *A* of a real network and decides statistical significance at which the partition for a certain value $k=k_{min}$ can represent the minimum balanced coloring of the real network. To decide this value, we employ a method which compares the correction cost, $\psi (k)$, of the real network with that of several randomized networks, which preserve the degree sequence. Next we outline the process by which we shuffle the entries of the weighted matrix *A* to produce these randomized networks.

1. Choose four integers *i*, *j*, *l*, *m* randomly from 1 to *N*, such that $i\ne j\ne l\ne m$.
2. Swap the following entries:
  12 $$\begin{aligned} \begin{aligned} A_{ij} \leftrightarrow A_{il}\\ A_{ji} \leftrightarrow A_{li}\\ A_{mj} \leftrightarrow A_{ml}\\ A_{jm} \leftrightarrow A_{lm} \end{aligned} \end{aligned}$$
3. Repeat steps (1) and (2) $n_s$ times until the network is sufficiently shuffled.

Using this algorithm we build a data set of 100 randomized networks for each structure we want to analyze. Then we compute the correction cost for each randomized network in the database. Finally, we calculate the mean value and the standard deviation for the obtained correction cost. At this point, we define $k_{min}$ as the lowest statistically significant value *k* for which the actual correction cost goes below three standard deviations of the mean randomized correction cost.

**Figure 4:**
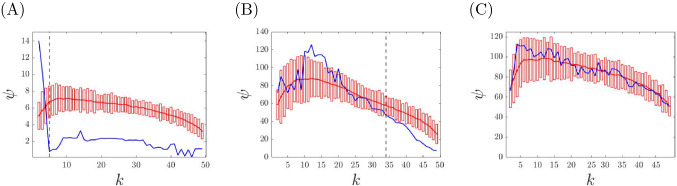
We plot the correction cost (blue curve) with statistical significance (red bars) and the mean of the randomized correction cost (red curve) as described above. (**A**–**C**) Synthetic network depicted in Fig. 2. (**A**) Type I perturbation with $\epsilon =10^{-1}$. (**B**) Type I perturbation with $\epsilon =10^{0}$. (**C**) Type II perturbation with $\epsilon =10^{0}$. Based on the size of the perturbations we have that either (**A**) the true cluster partition is recovered, or (**B**) a subset of the symmetries are recovered, or (**C**) all symmetries are lost to a statistical significant level.

**Figure 5:**
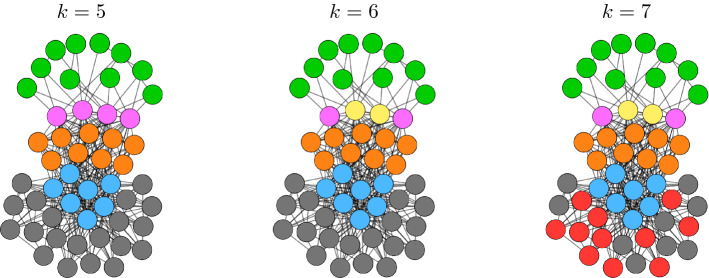
The evolution of the $N=50$ node network with $k^*=5$ true clusters depicted in Fig. 2. We apply a small perturbation (Type I, $\epsilon =10^{-5}$). With each increase of *k*, the general structure of the partition is preserved, however existing clusters are broken.

**Figure 6:**
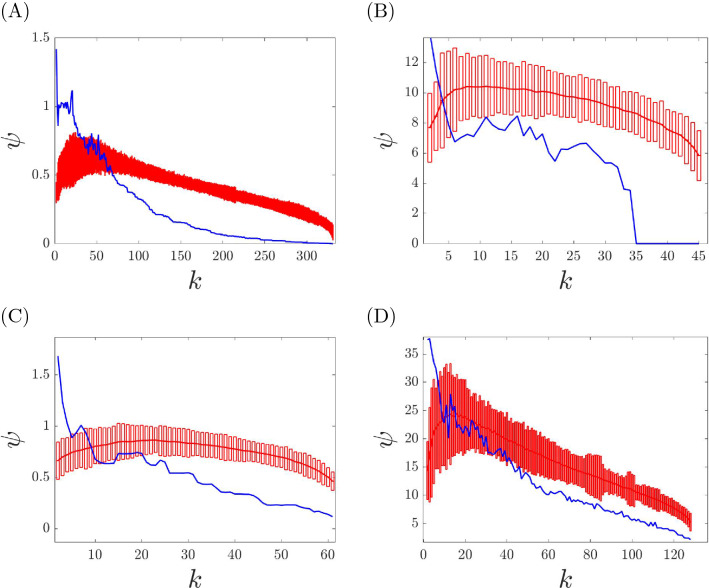
Correction cost (blue curves) with statistical significance analysis (red bars) for real world networks. (**A**) US Air network. (**B**) Freeman’s researcher network. (**C**) Stock market network. (**D**) Brain network.

Given an unweighted network, there is an ordered set (a lattice^33^) of exact equitable partitions, from the minimum balanced coloring (the equitable partition with the fewest clusters) to the partition in which each node is in a cluster by itself. In the case of weighted networks, we are typically interested in those equitable partitions (and the corresponding dynamical communities) that are statistically significant. The first important observation is that in many of the synthetic and real networks we have tested, we see that the correction cost is low to a statistically significant level for all $k \ge k_{min}$. We conclude that all of the returned solutions for $k = k_{min},\ldots ,N-1$ are statistically significant nearly equitable partitions. Also, with an abuse of language we call the partition obtained at $k=k_{min}$ the minimum balanced coloring of the weighted network. The second important observation is that in perturbed synthetic networks, $k_{min}$ is found to increase with the level of noise. A larger level of noise corresponds to more equivalence relations being destroyed, and so to a minimum balance coloring with more dynamical communities. This is seen in Fig. 4 where a synthetic network is modified with increasing level of noise. For a small level of noise (Fig. 4A) we find that $k_{min}=k^*$, i.e., the true number of clusters of the synthetic network with no noise; for intermediate level of noise (Fig. 4B) we obtain a value of $k_{min}>k^*$, and for large level of noise (Fig. 4C) we find no statistically significant *k*, indicating that no equivalence relation is preserved. Figure 6 shows the case of a real network, which appears to resemble a situation of intermediate noise, for which statistically significant equivalence relations can be detected for $k\ge k_{min}$. The third important observation is that similarly to the case of unweighted networks, we often see a *structure* in the solution obtained when *k* is increased from $k_{min}$ to $N-1$, namely the solution observed at $k+1$ is equal to the solution obtained at *k* after one cluster is broken into two different clusters. This can be seen in Fig. 5, which shows the obtained cluster partitions for $k=5,6,7$ for the perturbed synthetic network in Fig. 2 ($k^*=5$). As *k* is increased, the general structure of the partition is preserved, however dynamical communities are progressively broken. In particular, from $k=5$ to $k=6$ the dynamical community of four purple nodes splits into two smaller dynamical communities (purple nodes and yellow nodes for $k=6$). Similarly, from $k=6$ to $k=7$ the dynamical community of gray nodes splits into two smaller ones shown as gray and red in the network representation for $k=7$. This trend will continue until $k=N-1$.

## Real data sets

We apply our method to a number of real network data sets, including one social network^34^, one biological network, one air traffic network and one stock market network^35,36^. The temporal Freeman’s researcher social network describes the time evolution of personal relationships among $N=46$ researchers, where an edge value describes the strength of the relationship. A weight of 4 describes a close personal friend and going down from there, a weight of 0 means the person is unknown. The temporal network includes two *snapshots*, the first one describes relationship data from the beginning of the study and the second network is after the study. The stock market network is weighted, contains $N=62$ nodes, and comes from the correlation of long-returns from 62 different stocks. The US Air network is an undirected, weighted network. It contains $N=332$ nodes where each node represents an airport and edges represent the number of direct flights between airports. The biological network is a brain network taken from the Human Connectome Project^45^. The network is weighted, bidirectional and contains N=129 nodes. 

We have checked for statistically significant dynamical communities in each of these data sets. The result of this study are illustrated in Fig. 6: panel (A) is for the US Air network, panel (B) is for the Freeman’s researcher network (correlating to the end of the study), panel (C) is for the Stock market network, panel (D) is for subject #1 of the brain network dataset. Although we have tested multiple subjects from the brain network dataset, the correction cost plots for all subjects look qualitatively similar. The Freeman’s researcher network with integer weights has true equitable partitions for $k^* \ge 35$, as can be seen from the correction cost $\psi$ becoming zero. However, statistically significant dynamical communities are also found for $k < k^*$. All the other networks examined, both those with integer and non-integer weights, do not have true cluster partitions that are found at $\psi =0$.

As seen in Fig. 5, we typically observe that cluster partitions break down in an orderly way as *k* is increased, preserving the general structure of the partition. This is not only true for perturbed synthetic networks, but also for many real networks, as can be seen from Fig. 2 of the SI. The tendency for strongly symmetric nodes to be placed in the same cluster together in multiple partitions for different values of *k* can be seen in several networks.

**Figure 7:**
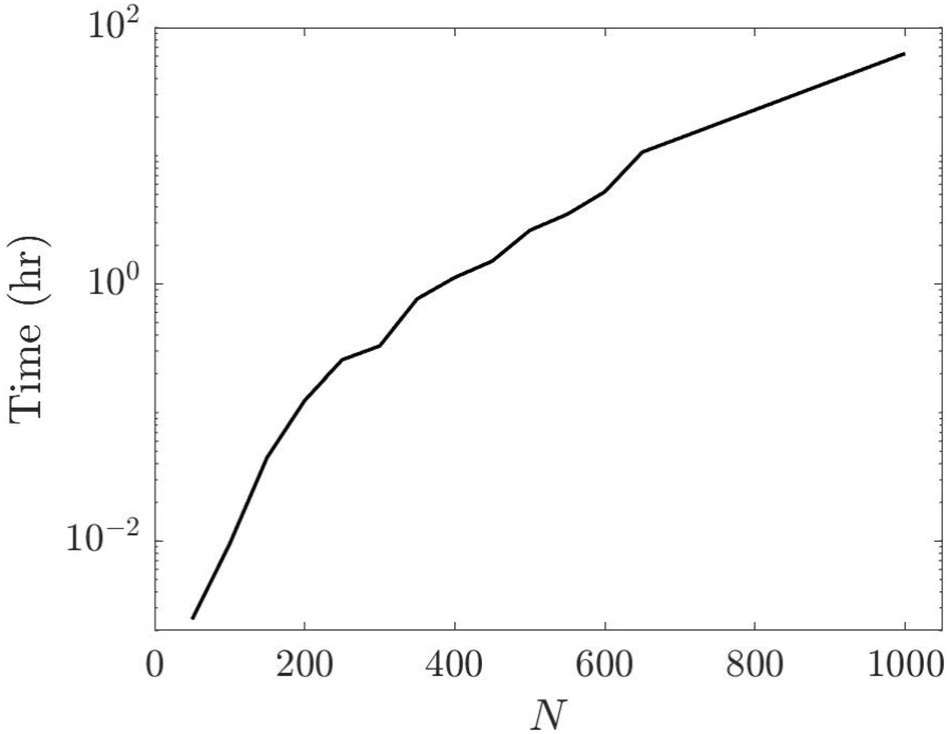
Time (h) versus network size (N). As the number of nodes increases, the calculation time increases rapidly.

In order to quantify the speed of this algorithm, we conduct a simple experiment where we calculate all cluster partitions from $k=2\ldots N-1$ for Erdos-Renyi networks with a varying number of nodes *N*, and time how long it takes . For our networks, we use sparse undirected graphs with weights randomly drawn from a uniform distribution between 0 and 1. We plot our results in Fig. 7

.

## Comparison with community structure

Our method to detect dynamical communities in weighted networks can be closely compared to methods to detect community structure. Community structure in networks occurs when there are groups of nodes that are densely connected together, and when these groups of nodes are sparsely connected to other groups. Methods based on community structure are not designed to predict approximate dynamical consensus and synchronization, but simply  look for modules with dense connectivity inside each module and sparse connectivity between modules. . Below, we illustrate that dynamical communities are much more accurate in predicting dynamical consensus than community structure, when applied to several real network examples.

For the purpose of this paper, we use Newman’s community structure algorithm^37^, where we can recover the hierarchical breakdown of the communities, so we can get a partition for all $k=2\ldots (N-1)$. We compare our method of detecting dynamical communities with that for community structure in terms of our ability to predict the consensus dynamics. We take the dynamics of a network to be described by Eq. (9) (where $\rho$ is large enough, so *A* is Hurwitz). We integrate Eq. (9) from $t=0$ to the settling time $t=-4/\Lambda$, where $\Lambda$ is the largest real eigenvalue of the matrix *A*. We apply *k-means* to *y*(*t*), where we specify *k* to be $k=2\ldots N-1$. We now have a cluster partition pertaining to the transient dynamics for all values *k*. Using this cluster partition as a benchmark to represent the *approximate dynamical clusters*, we use the Jaccard index to compare the similarity of this partition with that of our dynamical communities and community structure. The larger the Jaccard coefficient, ${\mathcal {J}}$, the more similar the cluster partitions are (with ${\mathcal {J}}=1$ indicating a perfect match).

In our analysis we consider the following real networks from the literature:

(A) The journal and magazine network^38^ is undirected, weighted, and contains $N=124$ nodes. Each node represents a journal or magazine and an edge represents the number of people who read them both.
(B) The Train Bombing social network^39^ is undirected, weighted and contains $N=64$ nodes. Each node represents a terrorist involved in the 2004 train bombing in Madrid, and an edge between them signifies a contact between two terrorists.
(C) The Kangaroo network is undirected, weighted and contains $N=17$ nodes. The network describes interactions between free-ranging eastern grey kangaroos in the Nadgee Nature Reserve in New South Wales, Australia. A node represents a kangaroo and an edge represents an interaction between them. The edge weights denote the number of interactions.
(D) The Southern Women Club social network^40^ is labeled, and contains $N=18$ nodes. The data comes from observed attendance of fourteen social events, where an edge between two nodes (subjects) represents the number of social events attended in common.
(E) The stock market network is weighted and undirected and contains $N=62$ nodes. The network is obtained from the analysis of temporal correlations among the time-series of stocks in the New York Exchange Market between January 2012 and December 2014.
(F) The IEEE 118-bus system^41^ is undirected and unweighted (weights between 0 and 1), it is an approximated representation of the U.S. Midwest Electric Power system as of December 1962.

**Figure 8:**
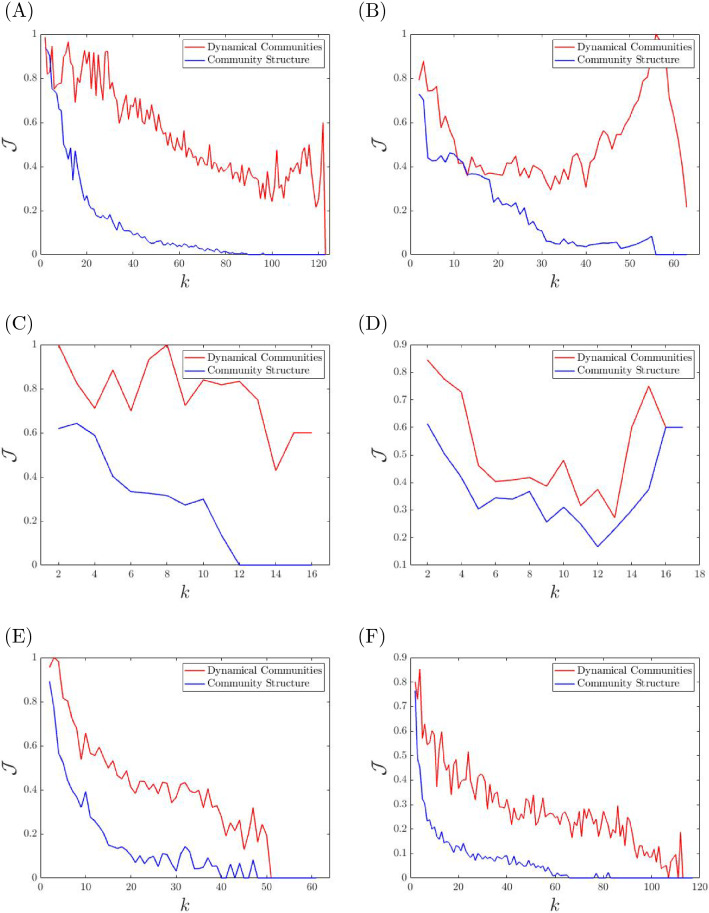
We plot the Jaccard index versus the number of clusters *k*, for both our dynamical communities (in red) and community structure (in blue). We plot the results for several real world networks, and see a strong advantage of using dynamical communities over community structure. The networks considered here are: (**A**) Journal and magazine^38^ (**B**) Train bomber^39^ (**C**) Kangaroo (**D**) Southern Women^40^ (**E**) Stock market (**F**) Power grid.

Figure 8 shows the Jaccard index versus the number of clusters *k*, for both our dynamical communities (in red) and community structure^37^ (in blue) when applied to the previously described real networks (A)–(F). For all the networks and for all values of *k* we see that our method based on dynamical communities consistently outperforms community structure in predicting synchronized behavior.

## Conclusions

In most real networks, node-node couplings are rarely characterized by identical strengths; in fact, weighted networks provide the most general paradigm to model interactions occurring in a complex system. In this work, we faced the problem of characterizing approximate cluster partitions and their ‘dynamical communities’ in weighted networks. We proposed a method based on calculation of a correction cost, namely a parameter quantifying how much the network has to be modified to obtain an exact cluster partition, and a statistical significance test to determine a minimum balanced coloring. Remarkably, our method retrieves the exact cluster partitions when applied to unweighted networks, as in that case the correction cost vanishes. Our variation to the BH algorithim for finding dynamical communities is not computationally demanding. It is not comparable to the speed of Newman’s fast algorithim, however, can still yield reasonable calculation speeds for small to moderately sized networks. If a test for statistical significance is desired (which includes calculating the correction cost over several random samples), the speed of the calculations decreases drastically with the size of the network.

Early studies had pointed out that the emergence of coordinated motion of clusters of nodes in unweighted or labeled graphs is only possible when the clusters form an equitable partition^2,7,8,10,15,23^. Methods to retrieve all the possible equitable partitions of a given network have been developed in^33,42^. Here we extend these concepts to the realm of weighted networks, for which equivalences between nodes may be satisfied approximately rather than exactly. The analysis of the correction cost in both synthetic and real networks reveals important features of dynamical communities. The correction is typically seen to change gradually as the number of clusters increases, with clusters breaking into groups of nodes of lower cardinality. Dynamical communities have been also considered in^43^, although in a different perspective. In^43^, a novel definition of quasi-symmetries relying on structural equivalence rather than the invariance of a particular topological property has been proposed based on the dynamical behavior of the Kuramoto-Saguchi model associated to the network nodes. However, in this case, the fact that nodes displaying similar states are almost symmetrical has to be considered as an a priori assumption rather than the result of structures with underlying similar patterns of connectivity. On the contrary, the notion of correction cost allows one to account for the similarity between different  interaction topologies, measuring the perturbation needed to transform one adjacency matrix into another.

Quite importantly, the dynamical communities considered in our work profoundly differ from network communities^22^ that account for modules with dense connections within the members of each module but sparse connections between members of different modules. In contrast with network communities, dynamical communities identify nodes that will produce approximately the same dynamical time evolution. Consequently, the presence of dynamical communities directly impacts the dynamics emerging from the network. For instance, our results show that nodes from the same dynamical community (from different dynamical communities) tend to display similar (different) consensus and synchronization dynamics and for the case of oscillatory chaotic dynamics, dynamical communities are seen to produce approximate cluster synchronization. Our work, therefore, paves the way towards the identification of relationships among the nodes that characterize a far from trivial interplay between dynamics and structure. For example, it may help uncover patterns of synchronous dynamics that may emerge in a network from knowledge of the network structure, even when very little is known about the dynamics itself. We expect that our methodology will find application in diverse fields, as we see from our analysis of real data sets that statistically significant nearly equivalent cluster partitions and dynamical communities are present in a large variety of social, biological, and technological networks (though not in all these networks.) Our approach could also be extended to the case of multilayer networks^9,44^.

For all the real networks examined, we see that our method based on dynamical communities consistently outperforms community structure in predicting synchronized behavior. Our work provides a unique perspective into the hidden relationship between network structure and network dynamics. We show that the analysis of the structure of a given network provides insight into the patterns of synchronous dynamics that may emerge in the network, even if very little is known about the dynamics itself. Our approach provides a fundamental advantage in understanding the dynamics of complex heterogeneous systems from different areas of biology and of the social sciences.

## Supplementary Information

Supplementary Information.

## Data Availability

The code used in this paper to find dynamical communities in weighted networks can be found here: https://github.com/chadnathe/Dynamical-Communities.gitGitHub Repository.
