# Supplementary material for: Looking beyond community structure leads to the discovery of dynamical communities in weighted networks
Source: Sci Rep. 2022 Mar 16;12:4524. doi: 10.1038/s41598-022-08214-z (PMC8927123; doi:10.1038/s41598-022-08214-z)
Supplement: Supplementary file 1 — Supplementary Information. [file 41598_2022_8214_MOESM1_ESM.pdf]

Supplementary Information

Looking Beyond Community Structure Leads to the Discovery of Dynamical Communities in Weighted Networks

Chad Nathe,[1] Lucia Valentina Gambuzza,[2] Mattia Frasca,[2] and Francesco Sorrentino[1]

[1] *Mechanical Engineering Department, University of New Mexico, Albuquerque, NM 87131*

[2] *Department of Electrical, Electronics and Computer Science Engineering, University of Catania, Catania, Italy.*

I. A DYNAMICAL APPROACH TO IDENTIFY NEARLY EQUITABLE CLUSTERS

As an alternative to using the modified BH algorithim to generate cluster partitions, we propose another method capable of generating cluster partitions for each value of $k = 2, \ldots, N-1$. If we begin with a matrix, A_0, which describes the weighted topology of a network with a main diagonal of zeros, we can stabilize the matrix by performing the replacement,

$$\tilde{A} = A_0 - \rho I \tag{1}$$

where ρ is described by,

$$\rho = \lambda + \sigma. \tag{2}$$

Here, λ is the largest real eigenvalue of the matrix A_0, and σ is a stability controlling parameter $\infty < \sigma < 0$. We can now use the (stable) $\tilde{A}$ matrix to write the steady-state solution as:

$$x^{ss} = -\tilde{A}^{-1}\delta \tag{3}$$

By varying σ, we will get different x^{ss} solutions. Using this property, we see clusters of state variables emerge as σ is varied (and correspondingly the stability property of the equilibrium point x^{ss} changes from marginally stable to stable). In Fig. 1 we plot x^{ss} versus σ for the synthetic network depicted in Fig. 2A of the main text. Curves are colored consistently with the true cluster partition.

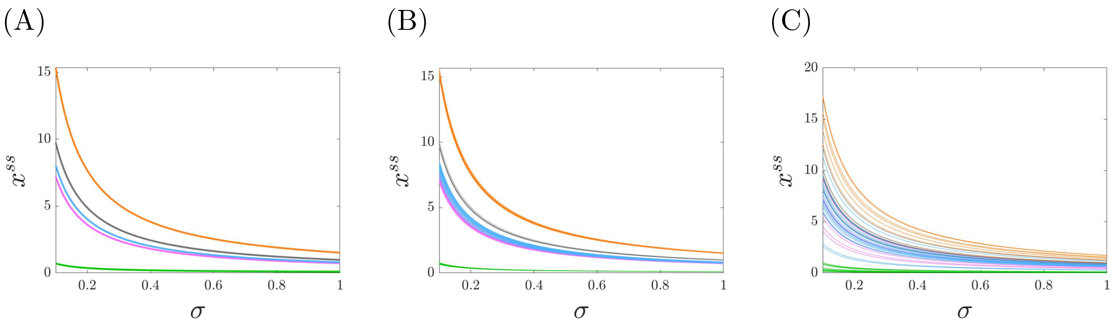

FIG. 1. x^{ss} versus σ with curves colored corresponding to the true cluster partition. (A) Unperturbed network. (B) Type I perturbation, $\epsilon = 10^{-1}$. (C) Type I perturbation, $\epsilon = 10^{0}$

Note that in Fig. 1 we are not plotting the dynamical evolution of the nodes. Instead we are plotting the steady states x^{ss} as a function of σ. For the unperturbed network, all curves belonging to the same cluster will be equivalent. As the perturbation increases, these curves will begin to separate. To create a cluster partition using this data, we can apply *k-means* to these curves and specify our desired k.

Now that we have an alternative method to create cluster partitions for $k = 2 \ldots N-1$, we propose a method alternative to the correction cost in order to determine the proper value k which represents the nearly equitable cluster partition.

Assuming a synthetic network with true clusters, from knowledge of the matrix A and of the true clusters, we can also write the equation of the quotient network,

$$\dot{y}(t) = -Qy(t) + \beta, \tag{4}$$

where $Q = (E^T E)^{-1} E^T A E$, $\beta = (E^T E)^{-1} E^T \delta$ and E is the indicator matrix, with steady state

$$y^{ss} = Q^{-1}\beta. \tag{5}$$

We now consider a transformation from the x coordinates into the y irreducible coordinates, where $y(t) = Tx(t)$ and $T = [T_{par}^T \;\; T_{orth}^T]^T$ is the orthogonal transformation matrix defined in previous work, see e.g.,[1,2]. We can then write,

$$\begin{aligned} y(t)_{par} &= T_{par} x(t) \\ y(t)_{orth} &= T_{orth} x(t) \end{aligned} \tag{6}$$

and,

$$\|x(t)\|^2 = \|y(t)\|^2 = \|y(t)_{par}\|^2 + \|y(t)_{orth}\|^2 \tag{7}$$

In an unweighted or labeled network with true clusters, given the T_{par} matrix is constructed according to the true cluster partition, the $\|y(t)_{orth}\|^2$ term in Eq. (7) will go to 0. Using the method described above, we are able to create T_{par} matrices correlating to each cluster partition. Using Eqs. (6) and (7), we are able to calculate $\|y(t)_{orth}\|$. For real networks, or perturbed synthetic networks, the closer the T_{par} matrix is to the underlying symmetries of A, the closer $\|y(t)_{orth}\|$ will be to 0. By making $\|y(t)_{orth}\|$ a function of k, we have the ability, similar to the correction cost method, to decide statistical significance as described in the main text.

II. ADDITIONAL CLUSTER PARTITION ANALYSIS

Here we show the cluster partition evolution as k is increased, starting from k_{min} for the Southern Women Club network and Freeman Researcher network (a similar plot for one of the brain networks is shown in Fig. 4 of the main text.) In both cases, the general structure of the partition is preserved and new clusters are formed when nodes break off from existing clusters. However, with the Freeman Research network we sometimes see that nodes may join existing clusters when $k \leftarrow k+1$, and can also revert back when k is increased.

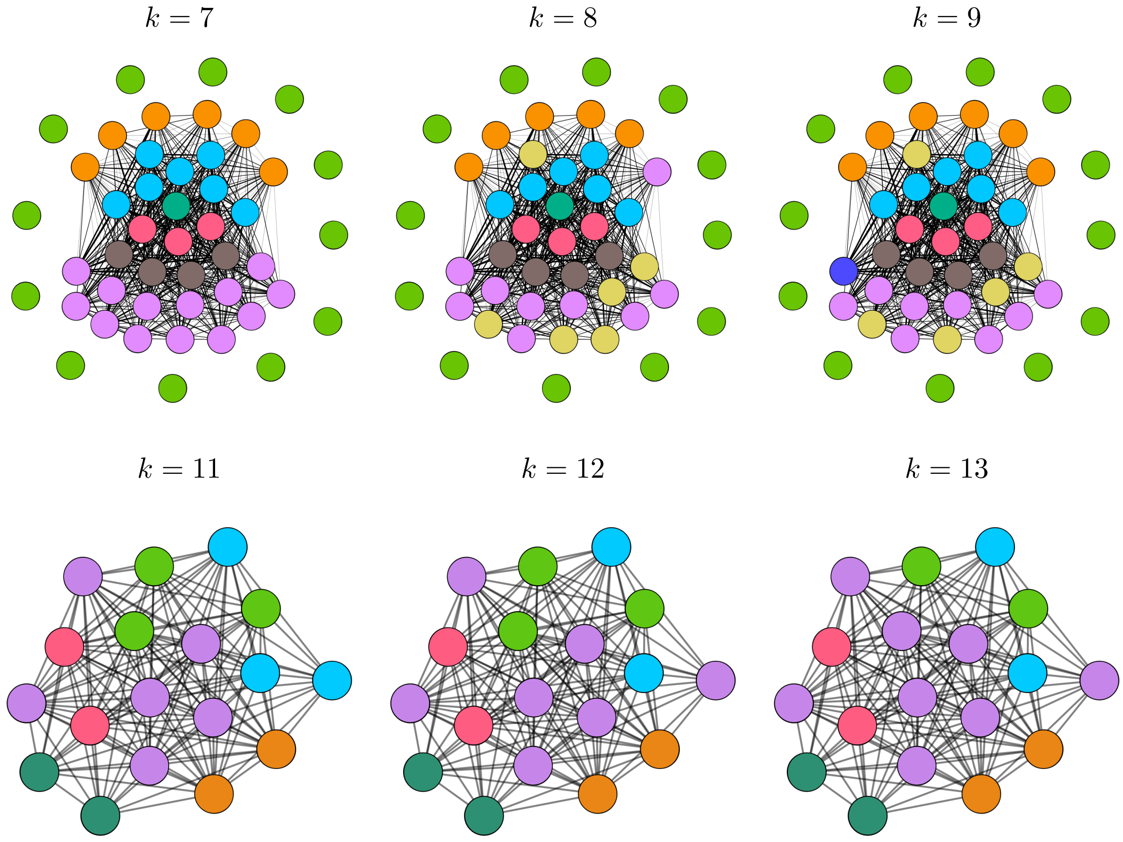

FIG. 2. Cluster partitions at k_{min}, $k_{min}+1$, $k_{min}+2$. Top panel, Freeman researcher network (time 2, end of study). Bottom panel, Southern Women Club social network, where all clusters containing only one node are colored purple.

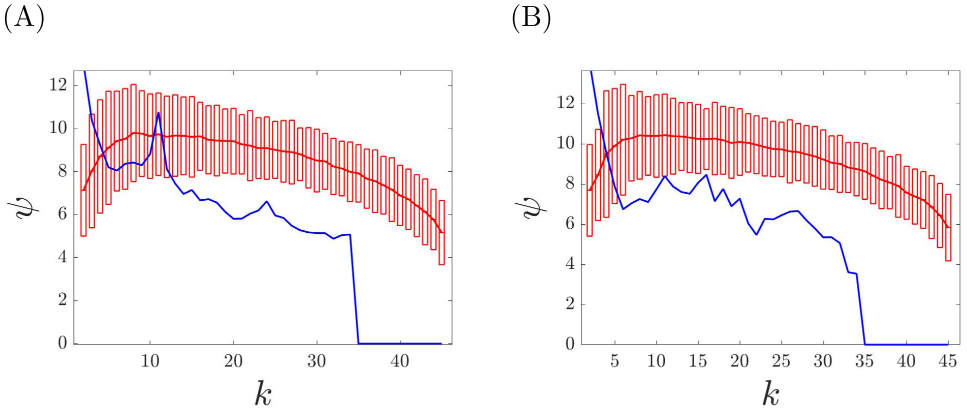

FIG. 3. Temporal Freeman Researcher Network. Correction cost (blue curve) and statistical significance (red bars) versus k. (A) Network at time 1 (beginning of study). (B) Network at time 2 (end of study).

REFERENCES

[1] Pecora, L. M., Sorrentino, F., Hagerstrom, A. M., Murphy, T. E., and Roy, R., "Cluster synchronization and isolated desynchronization in complex networks with symmetries," Nature Communications **5** (2014).

[2] Sorrentino, F., Pecora, L. M., Hagerstrom, A. M., Murphy, T. E., and Roy, R., "Complete characterization of stability of cluster synchronization in complex dynamical networks," Science Advances **2** (2016).
